# Bis(3-bromo­methyl-2-meth­oxy-1-naphth­yl)methane

**DOI:** 10.1107/S1600536808027529

**Published:** 2008-08-30

**Authors:** Jie-Wei Luo, Li Zhou, Rui He, Gui-Yu Wang, Da-Bin Qin

**Affiliations:** a School of Chemistry and Chemical Engineering, China West Normal University, Nanchong 637002, People’s Republic of China

## Abstract

The title compound, C_25_H_22_Br_2_O_2_, crystallizes with two mol­ecules in the asymmetric unit. In each independent mol­ecule, the two naphthalene ring systems are nearly perpendicular to one another, with dihedral angles of 85.6 (1) and 86.2 (1)°. The crystal structure is stabilized by C—H⋯π inter­actions, and inter- and intra­molecular C—H⋯O and C—H⋯Br hydrogen bonds.

## Related literature

For the synthesis, see: Failla *et al.* (1993 ▶). For a related structure, see: Liu *et al.* (2006 ▶). For related literature, see: Fonge *et al.* (2007 ▶); Georghiou *et al.* (1996 ▶); Haynes *et al.* (2006 ▶); Kano *et al.* (1995 ▶); Kondekar & Potlis (1973 ▶).
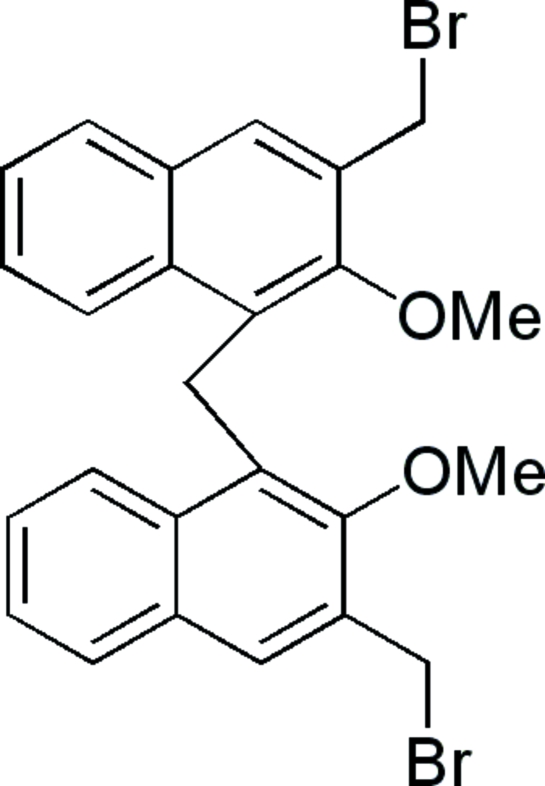


## Experimental

### 

#### Crystal data

C_25_H_22_Br_2_O_2_
*M* *_r_* = 514.25
Triclinic, 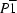

*a* = 10.6812 (3) Å
*b* = 11.1429 (4) Å
*c* = 18.4311 (6) Å
α = 99.327 (1)°
β = 98.326 (1)°
γ = 96.296 (1)°
*V* = 2121.84 (12) Å^3^
*Z* = 4
Mo *K*α radiation
μ = 3.84 mm^−1^
*T* = 153 (2) K
0.54 × 0.36 × 0.34 mm

#### Data collection

Rigaku R-AXIS RAPID diffractometer
Absorption correction: multi-scan (*SADABS*; Siemens, 1996 ▶) *T*_min_ = 0.208, *T*_max_ = 0.271
20944 measured reflections
9619 independent reflections
6284 reflections with *I* > 2σ(*I*)
*R*_int_ = 0.046

#### Refinement

*R*[*F* ^2^ > 2σ(*F* ^2^)] = 0.052
*wR*(*F* ^2^) = 0.209
*S* = 1.01
9619 reflections
528 parameters
H-atom parameters constrained
Δρ_max_ = 1.01 e Å^−3^
Δρ_min_ = −1.48 e Å^−3^

### 

Data collection: *RAPID-AUTO* (Rigaku/MSC 2004 ▶); cell refinement: *RAPID-AUTO*; data reduction: *RAPID-AUTO*; program(s) used to solve structure: *SHELXS97* (Sheldrick, 2008 ▶); program(s) used to refine structure: *SHELXL97* (Sheldrick, 2008 ▶); molecular graphics: *SHELXTL* (Sheldrick, 2008 ▶); software used to prepare material for publication: *SHELXTL*.

## Supplementary Material

Crystal structure: contains datablocks global, I. DOI: 10.1107/S1600536808027529/pv2087sup1.cif

Structure factors: contains datablocks I. DOI: 10.1107/S1600536808027529/pv2087Isup2.hkl

Additional supplementary materials:  crystallographic information; 3D view; checkCIF report

## Figures and Tables

**Table 1:** Hydrogen-bond geometry (Å, °)

*D*—H⋯*A*	*D*—H	H⋯*A*	*D*⋯*A*	*D*—H⋯*A*
C12—H12*A*⋯Br1^i^	0.99	2.91	3.867 (6)	164
C37—H37*B*⋯O2^ii^	0.99	2.56	3.384 (8)	141
C8—H8⋯O2	0.95	2.26	3.197 (7)	168
C11—H11*C*⋯Br1	0.98	2.86	3.575 (7)	131
C21—H21⋯O1	0.95	2.25	3.145 (7)	156
C33—H33⋯O4	0.95	2.32	3.244 (7)	164
C46—H46⋯O3	0.95	2.26	3.197 (7)	168
C5—H5⋯*Cg*4^iii^	0.95	2.63	3.511 (7)	155
C49—H49*B*⋯*Cg*7^iv^	0.98	2.73	3.505 (7)	136
C43—H43⋯*Cg*1	0.95	2.71	3.625 (7)	161

Symmetry codes: (i) 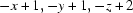; (ii) 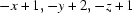; (iii) 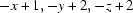; (iv) 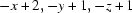. *Cg*1, *Cg*4 and *Cg*7 are the centroids of atoms C1–C4,C9,C10, C17–C22 and C26–C29,C34,C35, respectively.

**Fig. 1.**
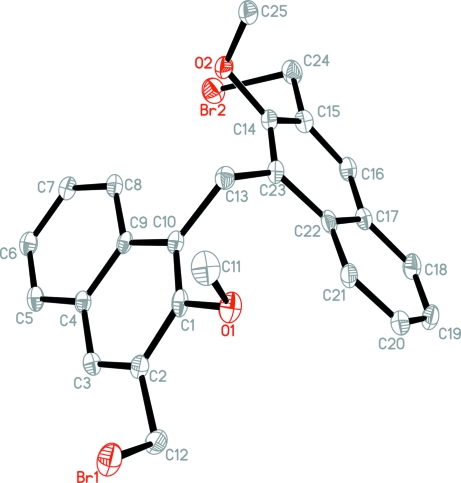
The molecular structure of the title compound, showing 30% probability displacement ellipsoids and the atomic numbering.

## supplementary crystallographic information

### Comment

Pamoic acid and its derivatives have attracted the attention of some scienctists (Kano *et al.*, 1995; Fonge *et al.*, 2007). The involved role has been performed in the pharmaceutical industry for the preparation of medicaments for the treatment of diseases (Haynes *et al.*, 2006). We have synthesized a derivative compound of pamoci acid according to the reported literature (Georghiou *et al.*, 1996; Kondekar & Potlis, 1973; Failla *et al.*, 1993). By reference to the crystal structure reported in the literature (Liu *et al.*, 2006), we report the crystal structure of the title compound, (I), in this paper.

The title compound crystallizes in the triclinic space group P-1 with two independent molecules in an asymmetric unit (Fig. 1). In each molecule, the two naphthalene ring systems are nearly perpendicular to one another. The dihedral angle between the C1—C10 and C14—C23 planes is 85.6 (1)° and that between the C26—C35 and C39—C48 planes is 86.2 (1)°. The crystal packing is stabilized by C—H···π interactions, intermolecular and intramolecular C—H···O and C—H···Br hydrogen bonds (Table 1).

### Experimental

The title compound was prepared according to the reported procedure of Failla *et al.* (1993). Colourless single crystals suitable for X-ray diffraction were obtained by recrystallization from dichloromethane.

### Refinement

H atoms were placed in calculated positions with C—H = 0.95–0.99 Å, and refined in riding mode with *U*_iso_(H) = 1.2*U*_eq_(C). The largest residual electron density was located in the close proximity of Br atom and was deemed meaningless.

### Crystal data

**Table:**

C_25_H_22_Br_2_O_2_	*Z* = 4
*M**_r_* = 514.25	*F*(000) = 1032
Triclinic, *P*1	*D*_x_ = 1.610 Mg m^−^^3^
*a* = 10.6812 (3) Å	Mo *K*α radiation, λ = 0.71073 Å
*b* = 11.1429 (4) Å	Cell parameters from 14353 reflections
*c* = 18.4311 (6) Å	θ = 3.2–27.5°
α = 99.327 (1)°	µ = 3.84 mm^−^^1^
β = 98.326 (1)°	*T* = 153 K
γ = 96.296 (1)°	Block, colourless
*V* = 2121.84 (12) Å^3^	0.54 × 0.36 × 0.34 mm

### Data collection

**Table:**

Rigaku R-AXIS RAPID diffractometer	9619 independent reflections
Radiation source: Rotating Anode	6284 reflections with *I* > 2σ(*I*)
graphite	*R*_int_ = 0.046
ω scans	θ_max_ = 27.5°, θ_min_ = 3.2°
Absorption correction: multi-scan (*SADABS*; Siemens, 1996)	*h* = −13→12
*T*_min_ = 0.208, *T*_max_ = 0.271	*k* = −14→14
20944 measured reflections	*l* = −23→23

### Refinement

**Table:**

Refinement on *F*^2^	Secondary atom site location: difference Fourier map
Least-squares matrix: full	Hydrogen site location: inferred from neighbouring sites
*R*[*F*^2^ > 2σ(*F*^2^)] = 0.052	H-atom parameters constrained
*wR*(*F*^2^) = 0.209	*w* = 1/[σ^2^(*F*_o_^2^) + (0.1*P*)^2^ + 9.88*P*] where *P* = (*F*_o_^2^ + 2*F*_c_^2^)/3
*S* = 1.01	(Δ/σ)_max_ = 0.001
9619 reflections	Δρ_max_ = 1.01 e Å^−^^3^
528 parameters	Δρ_min_ = −1.48 e Å^−^^3^
0 restraints	Extinction correction: *SHELXL97* (Sheldrick, 2008), Fc^*^=kFc[1+0.001xFc^2^λ^3^/sin(2θ)]^-1/4^
Primary atom site location: structure-invariant direct methods	Extinction coefficient: 0.0078 (8)

### Special details

**Table:**

Geometry. All e.s.d.'s (except the e.s.d. in the dihedral angle between two l.s. planes)are estimated using the full covariance matrix. The cell e.s.d.'s are takeninto account individually in the estimation of e.s.d.'s in distances, anglesand torsion angles; correlations between e.s.d.'s in cell parameters are onlyused when they are defined by crystal symmetry. An approximate (isotropic)treatment of cell e.s.d.'s is used for estimating e.s.d.'s involving l.s. planes.
Refinement. Refinement of *F*^2^ against ALL reflections. The weighted *R*-factor *wR* and goodness of fit *S* are based on *F*^2^, conventional *R*-factors *R* are based on *F*, with *F* set to zero for negative *F*^2^. The threshold expression of *F*^2^ > σ(*F*^2^) is used only for calculating *R*-factors(gt) *etc*. and is not relevant to the choice of reflections for refinement. *R*-factors based on *F*^2^ are statistically about twice as large as those based on *F*, and *R*- factors based on ALL data will be even larger.

### Fractional atomic coordinates and isotropic or equivalent isotropic displacement parameters (Å^2^)

**Table:**

	*x*	*y*	*z*	*U*_iso_*/*U*_eq_	
Br1	0.68949 (6)	0.53790 (6)	0.94933 (4)	0.0416 (2)	
Br2	0.15252 (7)	1.26868 (6)	0.81324 (4)	0.0466 (2)	
O1	0.4066 (4)	0.6110 (3)	0.8623 (2)	0.0326 (9)	
O2	0.1981 (4)	0.9873 (4)	0.7210 (2)	0.0335 (9)	
C1	0.4635 (5)	0.7316 (5)	0.8845 (3)	0.0264 (11)	
C2	0.5619 (5)	0.7565 (5)	0.9475 (3)	0.0277 (11)	
C3	0.6255 (5)	0.8719 (5)	0.9705 (3)	0.0275 (11)	
H3	0.6919	0.8879	1.0125	0.033*	
C4	0.5947 (5)	0.9682 (5)	0.9333 (3)	0.0237 (10)	
C5	0.6642 (5)	1.0863 (5)	0.9536 (3)	0.0295 (12)	
H5	0.7345	1.1014	0.9933	0.035*	
C6	0.6342 (6)	1.1803 (5)	0.9181 (3)	0.0322 (12)	
H6	0.6831	1.2594	0.9328	0.039*	
C7	0.5298 (5)	1.1581 (5)	0.8595 (3)	0.0296 (12)	
H7	0.5083	1.2231	0.8345	0.036*	
C8	0.4587 (5)	1.0448 (5)	0.8378 (3)	0.0265 (11)	
H8	0.3881	1.0323	0.7983	0.032*	
C9	0.4892 (5)	0.9457 (5)	0.8736 (3)	0.0228 (10)	
C10	0.4211 (5)	0.8242 (5)	0.8502 (3)	0.0249 (11)	
C11	0.4446 (7)	0.5474 (6)	0.7968 (4)	0.0405 (15)	
H11A	0.4382	0.5978	0.7579	0.049*	
H11B	0.3885	0.4693	0.7790	0.049*	
H11C	0.5330	0.5316	0.8087	0.049*	
C12	0.5882 (6)	0.6603 (6)	0.9942 (4)	0.0340 (13)	
H12A	0.5059	0.6172	1.0012	0.041*	
H12B	0.6349	0.7010	1.0440	0.041*	
C13	0.3048 (5)	0.7976 (5)	0.7883 (3)	0.0279 (11)	
H13A	0.3281	0.8296	0.7442	0.034*	
H13B	0.2809	0.7076	0.7735	0.034*	
C14	0.1470 (5)	0.9488 (5)	0.7794 (3)	0.0303 (12)	
C15	0.0549 (5)	1.0166 (5)	0.8078 (3)	0.0292 (12)	
C16	−0.0010 (5)	0.9820 (5)	0.8653 (3)	0.0322 (12)	
H16	−0.0608	1.0291	0.8855	0.039*	
C17	0.0295 (5)	0.8775 (5)	0.8945 (3)	0.0299 (12)	
C18	−0.0321 (6)	0.8375 (6)	0.9509 (4)	0.0379 (14)	
H18	−0.0941	0.8830	0.9700	0.045*	
C19	−0.0049 (6)	0.7361 (6)	0.9784 (4)	0.0401 (15)	
H19	−0.0461	0.7118	1.0171	0.048*	
C20	0.0852 (6)	0.6667 (6)	0.9491 (4)	0.0358 (13)	
H20	0.1018	0.5937	0.9669	0.043*	
C21	0.1490 (5)	0.7032 (5)	0.8951 (3)	0.0304 (12)	
H21	0.2101	0.6558	0.8766	0.037*	
C22	0.1247 (5)	0.8112 (5)	0.8667 (3)	0.0289 (12)	
C23	0.1905 (5)	0.8540 (5)	0.8109 (3)	0.0279 (11)	
C24	0.1469 (6)	0.9104 (6)	0.6506 (3)	0.0379 (14)	
H24A	0.1588	0.8252	0.6535	0.046*	
H24B	0.1912	0.9374	0.6119	0.046*	
H24C	0.0556	0.9158	0.6384	0.046*	
C25	0.0176 (6)	1.1246 (6)	0.7760 (4)	0.0399 (15)	
H25A	−0.0643	1.1447	0.7906	0.048*	
H25B	0.0056	1.1049	0.7209	0.048*	
Br3	0.56974 (7)	0.93399 (6)	0.41869 (5)	0.0514 (2)	
Br4	0.75222 (7)	0.32634 (6)	0.74313 (5)	0.0537 (2)	
O3	0.8614 (4)	0.8227 (3)	0.4593 (2)	0.0326 (9)	
O4	0.9745 (3)	0.4825 (3)	0.6532 (2)	0.0258 (8)	
C26	0.7776 (5)	0.7143 (5)	0.4496 (3)	0.0261 (11)	
C27	0.6639 (5)	0.7017 (5)	0.3961 (3)	0.0297 (12)	
C28	0.5789 (6)	0.5975 (6)	0.3849 (3)	0.0330 (13)	
H28	0.5016	0.5900	0.3508	0.040*	
C29	0.6038 (5)	0.4998 (5)	0.4235 (3)	0.0305 (12)	
C30	0.5189 (6)	0.3890 (6)	0.4085 (4)	0.0366 (14)	
H30	0.4429	0.3807	0.3732	0.044*	
C31	0.5443 (6)	0.2931 (6)	0.4442 (4)	0.0435 (16)	
H31	0.4871	0.2185	0.4328	0.052*	
C32	0.6551 (6)	0.3061 (5)	0.4973 (4)	0.0392 (14)	
H32	0.6721	0.2399	0.5223	0.047*	
C33	0.7398 (6)	0.4133 (5)	0.5141 (3)	0.0316 (12)	
H33	0.8148	0.4199	0.5501	0.038*	
C34	0.7157 (5)	0.5142 (5)	0.4778 (3)	0.0263 (11)	
C35	0.8012 (5)	0.6269 (5)	0.4929 (3)	0.0252 (11)	
C36	0.9685 (6)	0.8085 (6)	0.4207 (4)	0.0404 (15)	
H36A	1.0170	0.7473	0.4398	0.048*	
H36B	1.0238	0.8873	0.4290	0.048*	
H36C	0.9375	0.7814	0.3672	0.048*	
C37	0.6374 (6)	0.8036 (6)	0.3563 (4)	0.0404 (15)	
H37A	0.5747	0.7714	0.3103	0.048*	
H37B	0.7171	0.8386	0.3416	0.048*	
C38	0.9161 (5)	0.6505 (5)	0.5552 (3)	0.0265 (11)	
H38A	0.9686	0.5830	0.5475	0.032*	
H38B	0.9693	0.7278	0.5526	0.032*	
C39	0.9041 (5)	0.5749 (5)	0.6763 (3)	0.0260 (11)	
C40	0.8609 (5)	0.5745 (5)	0.7451 (3)	0.0276 (11)	
C41	0.7961 (5)	0.6662 (5)	0.7709 (3)	0.0294 (12)	
H41	0.7642	0.6653	0.8163	0.035*	
C42	0.7752 (5)	0.7620 (5)	0.7320 (3)	0.0288 (12)	
C43	0.7128 (6)	0.8609 (6)	0.7612 (4)	0.0371 (14)	
H43	0.6834	0.8615	0.8075	0.045*	
C44	0.6945 (6)	0.9555 (6)	0.7231 (4)	0.0400 (15)	
H44	0.6532	1.0214	0.7434	0.048*	
C45	0.7362 (5)	0.9554 (5)	0.6550 (4)	0.0347 (13)	
H45	0.7240	1.0218	0.6294	0.042*	
C46	0.7948 (5)	0.8601 (5)	0.6244 (3)	0.0287 (12)	
H46	0.8222	0.8611	0.5777	0.034*	
C47	0.8148 (5)	0.7603 (4)	0.6617 (3)	0.0230 (10)	
C48	0.8787 (5)	0.6599 (5)	0.6321 (3)	0.0263 (11)	
C49	1.1086 (5)	0.5184 (5)	0.6793 (3)	0.0316 (12)	
H49A	1.1388	0.5881	0.6571	0.038*	
H49B	1.1548	0.4494	0.6649	0.038*	
H49C	1.1239	0.5423	0.7337	0.038*	
C50	0.8807 (6)	0.4714 (6)	0.7862 (4)	0.0361 (14)	
H50A	0.8744	0.4981	0.8393	0.043*	
H50B	0.9674	0.4490	0.7837	0.043*	

### Atomic displacement parameters (Å^2^)

**Table:**

	*U*^11^	*U*^22^	*U*^33^	*U*^12^	*U*^13^	*U*^23^
Br1	0.0362 (4)	0.0310 (3)	0.0661 (5)	0.0103 (3)	0.0220 (3)	0.0179 (3)
Br2	0.0432 (4)	0.0341 (3)	0.0608 (5)	0.0022 (3)	0.0023 (3)	0.0118 (3)
O1	0.033 (2)	0.0176 (18)	0.046 (2)	0.0013 (15)	0.0142 (18)	−0.0001 (17)
O2	0.030 (2)	0.036 (2)	0.032 (2)	−0.0096 (17)	0.0052 (17)	0.0057 (18)
C1	0.021 (3)	0.021 (2)	0.036 (3)	−0.002 (2)	0.014 (2)	−0.005 (2)
C2	0.023 (3)	0.028 (3)	0.033 (3)	0.001 (2)	0.009 (2)	0.007 (2)
C3	0.019 (3)	0.030 (3)	0.032 (3)	0.003 (2)	0.005 (2)	0.003 (2)
C4	0.020 (2)	0.024 (2)	0.026 (3)	0.001 (2)	0.008 (2)	−0.001 (2)
C5	0.023 (3)	0.024 (3)	0.038 (3)	−0.003 (2)	0.008 (2)	−0.003 (2)
C6	0.029 (3)	0.027 (3)	0.039 (3)	−0.003 (2)	0.010 (2)	0.004 (2)
C7	0.028 (3)	0.025 (3)	0.037 (3)	0.001 (2)	0.012 (2)	0.006 (2)
C8	0.022 (3)	0.027 (3)	0.030 (3)	0.000 (2)	0.009 (2)	0.001 (2)
C9	0.021 (2)	0.023 (2)	0.026 (3)	0.0016 (19)	0.014 (2)	0.001 (2)
C10	0.020 (2)	0.026 (3)	0.029 (3)	0.001 (2)	0.010 (2)	0.004 (2)
C11	0.043 (4)	0.028 (3)	0.047 (4)	0.005 (3)	0.011 (3)	−0.009 (3)
C12	0.030 (3)	0.035 (3)	0.044 (3)	0.008 (2)	0.013 (3)	0.017 (3)
C13	0.027 (3)	0.029 (3)	0.027 (3)	0.000 (2)	0.008 (2)	0.000 (2)
C14	0.023 (3)	0.036 (3)	0.030 (3)	−0.006 (2)	0.002 (2)	0.007 (2)
C15	0.025 (3)	0.024 (3)	0.037 (3)	0.003 (2)	0.006 (2)	0.001 (2)
C16	0.021 (3)	0.034 (3)	0.042 (3)	0.003 (2)	0.008 (2)	0.003 (3)
C17	0.018 (3)	0.035 (3)	0.036 (3)	0.001 (2)	0.004 (2)	0.005 (2)
C18	0.022 (3)	0.046 (4)	0.048 (4)	0.002 (3)	0.012 (3)	0.009 (3)
C19	0.030 (3)	0.050 (4)	0.038 (3)	−0.011 (3)	0.011 (3)	0.011 (3)
C20	0.027 (3)	0.037 (3)	0.041 (3)	−0.005 (2)	0.002 (3)	0.010 (3)
C21	0.020 (3)	0.030 (3)	0.041 (3)	−0.002 (2)	0.005 (2)	0.008 (2)
C22	0.020 (3)	0.030 (3)	0.031 (3)	−0.008 (2)	0.001 (2)	−0.001 (2)
C23	0.021 (3)	0.027 (3)	0.035 (3)	−0.002 (2)	0.007 (2)	0.004 (2)
C24	0.032 (3)	0.042 (3)	0.034 (3)	−0.012 (3)	0.003 (3)	0.004 (3)
C25	0.026 (3)	0.043 (4)	0.054 (4)	0.010 (3)	0.005 (3)	0.016 (3)
Br3	0.0450 (4)	0.0384 (4)	0.0830 (6)	0.0193 (3)	0.0279 (4)	0.0220 (4)
Br4	0.0385 (4)	0.0360 (4)	0.0887 (6)	−0.0014 (3)	0.0077 (4)	0.0240 (4)
O3	0.029 (2)	0.0239 (19)	0.044 (2)	−0.0045 (16)	0.0124 (18)	0.0047 (18)
O4	0.0193 (18)	0.0227 (18)	0.035 (2)	0.0035 (14)	0.0042 (15)	0.0030 (16)
C26	0.025 (3)	0.023 (2)	0.031 (3)	0.005 (2)	0.007 (2)	0.005 (2)
C27	0.027 (3)	0.029 (3)	0.035 (3)	0.006 (2)	0.008 (2)	0.007 (2)
C28	0.025 (3)	0.037 (3)	0.036 (3)	0.002 (2)	0.004 (2)	0.007 (3)
C29	0.024 (3)	0.030 (3)	0.034 (3)	−0.002 (2)	0.008 (2)	−0.003 (2)
C30	0.027 (3)	0.035 (3)	0.043 (3)	−0.005 (2)	0.003 (3)	0.001 (3)
C31	0.034 (3)	0.032 (3)	0.060 (4)	−0.010 (3)	0.006 (3)	0.006 (3)
C32	0.042 (4)	0.024 (3)	0.051 (4)	−0.001 (2)	0.013 (3)	0.004 (3)
C33	0.028 (3)	0.024 (3)	0.040 (3)	−0.001 (2)	0.008 (2)	0.001 (2)
C34	0.023 (3)	0.018 (2)	0.032 (3)	−0.0050 (19)	0.004 (2)	−0.009 (2)
C35	0.022 (3)	0.024 (3)	0.030 (3)	0.002 (2)	0.013 (2)	−0.001 (2)
C36	0.039 (3)	0.036 (3)	0.049 (4)	−0.005 (3)	0.024 (3)	0.007 (3)
C37	0.033 (3)	0.039 (3)	0.051 (4)	0.008 (3)	0.008 (3)	0.010 (3)
C38	0.019 (2)	0.030 (3)	0.030 (3)	0.000 (2)	0.009 (2)	0.002 (2)
C39	0.020 (3)	0.027 (3)	0.029 (3)	0.001 (2)	0.006 (2)	0.001 (2)
C40	0.025 (3)	0.029 (3)	0.024 (3)	−0.007 (2)	0.003 (2)	0.000 (2)
C41	0.021 (3)	0.035 (3)	0.031 (3)	0.001 (2)	0.008 (2)	0.001 (2)
C42	0.018 (2)	0.032 (3)	0.034 (3)	0.002 (2)	0.009 (2)	−0.003 (2)
C43	0.026 (3)	0.036 (3)	0.045 (4)	0.001 (2)	0.012 (3)	−0.008 (3)
C44	0.030 (3)	0.035 (3)	0.054 (4)	0.009 (3)	0.014 (3)	−0.007 (3)
C45	0.026 (3)	0.025 (3)	0.051 (4)	0.005 (2)	0.004 (3)	0.003 (3)
C46	0.023 (3)	0.027 (3)	0.038 (3)	0.004 (2)	0.013 (2)	0.001 (2)
C47	0.013 (2)	0.018 (2)	0.035 (3)	0.0007 (18)	0.006 (2)	−0.006 (2)
C48	0.019 (2)	0.023 (3)	0.038 (3)	−0.001 (2)	0.011 (2)	0.006 (2)
C49	0.022 (3)	0.029 (3)	0.041 (3)	0.001 (2)	0.006 (2)	0.000 (2)
C50	0.028 (3)	0.047 (4)	0.036 (3)	0.004 (3)	0.005 (2)	0.016 (3)

### Geometric parameters (Å, °)

**Table:**

Br1—C12	1.983 (6)	Br3—C37	1.971 (7)
Br2—C25	1.993 (7)	Br4—C50	1.977 (7)
O1—C1	1.384 (6)	O3—C26	1.392 (6)
O1—C11	1.432 (7)	O3—C36	1.441 (7)
O2—C14	1.382 (7)	O4—C39	1.388 (6)
O2—C24	1.430 (7)	O4—C49	1.434 (6)
C1—C10	1.378 (8)	C26—C35	1.377 (7)
C1—C2	1.417 (8)	C26—C27	1.428 (8)
C2—C3	1.357 (8)	C27—C28	1.360 (8)
C2—C12	1.506 (7)	C27—C37	1.479 (8)
C3—C4	1.407 (7)	C28—C29	1.422 (8)
C3—H3	0.9500	C28—H28	0.9500
C4—C5	1.403 (7)	C29—C30	1.412 (8)
C4—C9	1.426 (7)	C29—C34	1.420 (8)
C5—C6	1.364 (8)	C30—C31	1.373 (9)
C5—H5	0.9500	C30—H30	0.9500
C6—C7	1.406 (8)	C31—C32	1.401 (9)
C6—H6	0.9500	C31—H31	0.9500
C7—C8	1.367 (8)	C32—C33	1.380 (8)
C7—H7	0.9500	C32—H32	0.9500
C8—C9	1.420 (7)	C33—C34	1.427 (8)
C8—H8	0.9500	C33—H33	0.9500
C9—C10	1.432 (7)	C34—C35	1.432 (7)
C10—C13	1.527 (7)	C35—C38	1.522 (7)
C11—H11A	0.9800	C36—H36A	0.9800
C11—H11B	0.9800	C36—H36B	0.9800
C11—H11C	0.9800	C36—H36C	0.9800
C12—H12A	0.9900	C37—H37A	0.9900
C12—H12B	0.9900	C37—H37B	0.9900
C13—C23	1.514 (8)	C38—C48	1.518 (8)
C13—H13A	0.9900	C38—H38A	0.9900
C13—H13B	0.9900	C38—H38B	0.9900
C14—C23	1.377 (8)	C39—C48	1.370 (8)
C14—C15	1.414 (8)	C39—C40	1.411 (8)
C15—C16	1.375 (8)	C40—C41	1.358 (8)
C15—C25	1.489 (8)	C40—C50	1.493 (8)
C16—C17	1.411 (8)	C41—C42	1.398 (8)
C16—H16	0.9500	C41—H41	0.9500
C17—C18	1.413 (8)	C42—C47	1.419 (8)
C17—C22	1.426 (8)	C42—C43	1.422 (8)
C18—C19	1.355 (9)	C43—C44	1.375 (9)
C18—H18	0.9500	C43—H43	0.9500
C19—C20	1.412 (9)	C44—C45	1.390 (9)
C19—H19	0.9500	C44—H44	0.9500
C20—C21	1.373 (8)	C45—C46	1.376 (8)
C20—H20	0.9500	C45—H45	0.9500
C21—C22	1.422 (8)	C46—C47	1.418 (8)
C21—H21	0.9500	C46—H46	0.9500
C22—C23	1.441 (8)	C47—C48	1.447 (7)
C24—H24A	0.9800	C49—H49A	0.9800
C24—H24B	0.9800	C49—H49B	0.9800
C24—H24C	0.9800	C49—H49C	0.9800
C25—H25A	0.9900	C50—H50A	0.9900
C25—H25B	0.9900	C50—H50B	0.9900
			
C1—O1—C11	114.4 (4)	C26—O3—C36	112.8 (4)
C14—O2—C24	112.8 (4)	C39—O4—C49	111.6 (4)
C10—C1—O1	121.7 (5)	C35—C26—O3	120.9 (5)
C10—C1—C2	121.6 (5)	C35—C26—C27	122.0 (5)
O1—C1—C2	116.6 (5)	O3—C26—C27	116.9 (5)
C3—C2—C1	119.8 (5)	C28—C27—C26	119.1 (5)
C3—C2—C12	119.0 (5)	C28—C27—C37	121.1 (5)
C1—C2—C12	121.0 (5)	C26—C27—C37	119.8 (5)
C2—C3—C4	121.2 (5)	C27—C28—C29	121.2 (5)
C2—C3—H3	119.4	C27—C28—H28	119.4
C4—C3—H3	119.4	C29—C28—H28	119.4
C5—C4—C3	122.0 (5)	C30—C29—C34	119.7 (5)
C5—C4—C9	118.9 (5)	C30—C29—C28	120.9 (5)
C3—C4—C9	119.2 (5)	C34—C29—C28	119.3 (5)
C6—C5—C4	122.3 (5)	C31—C30—C29	121.1 (6)
C6—C5—H5	118.9	C31—C30—H30	119.4
C4—C5—H5	118.9	C29—C30—H30	119.4
C5—C6—C7	118.9 (5)	C30—C31—C32	119.5 (6)
C5—C6—H6	120.6	C30—C31—H31	120.2
C7—C6—H6	120.6	C32—C31—H31	120.2
C8—C7—C6	121.2 (5)	C33—C32—C31	121.1 (6)
C8—C7—H7	119.4	C33—C32—H32	119.4
C6—C7—H7	119.4	C31—C32—H32	119.4
C7—C8—C9	120.7 (5)	C32—C33—C34	120.4 (6)
C7—C8—H8	119.6	C32—C33—H33	119.8
C9—C8—H8	119.6	C34—C33—H33	119.8
C8—C9—C4	118.1 (5)	C29—C34—C33	118.0 (5)
C8—C9—C10	122.4 (5)	C29—C34—C35	119.4 (5)
C4—C9—C10	119.4 (5)	C33—C34—C35	122.5 (5)
C1—C10—C9	118.4 (5)	C26—C35—C34	118.6 (5)
C1—C10—C13	120.7 (5)	C26—C35—C38	120.3 (5)
C9—C10—C13	120.8 (5)	C34—C35—C38	121.2 (5)
O1—C11—H11A	109.5	O3—C36—H36A	109.5
O1—C11—H11B	109.5	O3—C36—H36B	109.5
H11A—C11—H11B	109.5	H36A—C36—H36B	109.5
O1—C11—H11C	109.5	O3—C36—H36C	109.5
H11A—C11—H11C	109.5	H36A—C36—H36C	109.5
H11B—C11—H11C	109.5	H36B—C36—H36C	109.5
C2—C12—Br1	113.0 (4)	C27—C37—Br3	111.7 (5)
C2—C12—H12A	109.0	C27—C37—H37A	109.3
Br1—C12—H12A	109.0	Br3—C37—H37A	109.3
C2—C12—H12B	109.0	C27—C37—H37B	109.3
Br1—C12—H12B	109.0	Br3—C37—H37B	109.3
H12A—C12—H12B	107.8	H37A—C37—H37B	107.9
C23—C13—C10	113.2 (5)	C48—C38—C35	112.8 (4)
C23—C13—H13A	108.9	C48—C38—H38A	109.0
C10—C13—H13A	108.9	C35—C38—H38A	109.0
C23—C13—H13B	108.9	C48—C38—H38B	109.0
C10—C13—H13B	108.9	C35—C38—H38B	109.0
H13A—C13—H13B	107.8	H38A—C38—H38B	107.8
C23—C14—O2	121.1 (5)	C48—C39—O4	119.6 (5)
C23—C14—C15	122.2 (5)	C48—C39—C40	123.4 (5)
O2—C14—C15	116.6 (5)	O4—C39—C40	117.0 (5)
C16—C15—C14	119.5 (5)	C41—C40—C39	118.7 (5)
C16—C15—C25	119.8 (5)	C41—C40—C50	121.1 (5)
C14—C15—C25	120.7 (5)	C39—C40—C50	120.2 (5)
C15—C16—C17	120.8 (5)	C40—C41—C42	121.4 (5)
C15—C16—H16	119.6	C40—C41—H41	119.3
C17—C16—H16	119.6	C42—C41—H41	119.3
C16—C17—C18	121.2 (5)	C41—C42—C47	119.9 (5)
C16—C17—C22	119.4 (5)	C41—C42—C43	121.1 (6)
C18—C17—C22	119.4 (5)	C47—C42—C43	119.0 (5)
C19—C18—C17	121.6 (6)	C44—C43—C42	120.6 (6)
C19—C18—H18	119.2	C44—C43—H43	119.7
C17—C18—H18	119.2	C42—C43—H43	119.7
C18—C19—C20	119.4 (6)	C43—C44—C45	120.4 (6)
C18—C19—H19	120.3	C43—C44—H44	119.8
C20—C19—H19	120.3	C45—C44—H44	119.8
C21—C20—C19	121.0 (6)	C46—C45—C44	120.6 (6)
C21—C20—H20	119.5	C46—C45—H45	119.7
C19—C20—H20	119.5	C44—C45—H45	119.7
C20—C21—C22	120.7 (6)	C45—C46—C47	120.8 (5)
C20—C21—H21	119.7	C45—C46—H46	119.6
C22—C21—H21	119.7	C47—C46—H46	119.6
C21—C22—C17	117.8 (5)	C46—C47—C42	118.6 (5)
C21—C22—C23	122.7 (5)	C46—C47—C48	122.4 (5)
C17—C22—C23	119.5 (5)	C42—C47—C48	119.0 (5)
C14—C23—C22	118.0 (5)	C39—C48—C47	117.3 (5)
C14—C23—C13	121.0 (5)	C39—C48—C38	121.9 (5)
C22—C23—C13	121.0 (5)	C47—C48—C38	120.8 (5)
O2—C24—H24A	109.5	O4—C49—H49A	109.5
O2—C24—H24B	109.5	O4—C49—H49B	109.5
H24A—C24—H24B	109.5	H49A—C49—H49B	109.5
O2—C24—H24C	109.5	O4—C49—H49C	109.5
H24A—C24—H24C	109.5	H49A—C49—H49C	109.5
H24B—C24—H24C	109.5	H49B—C49—H49C	109.5
C15—C25—Br2	110.0 (4)	C40—C50—Br4	111.0 (4)
C15—C25—H25A	109.7	C40—C50—H50A	109.4
Br2—C25—H25A	109.7	Br4—C50—H50A	109.4
C15—C25—H25B	109.7	C40—C50—H50B	109.4
Br2—C25—H25B	109.7	Br4—C50—H50B	109.4
H25A—C25—H25B	108.2	H50A—C50—H50B	108.0
			
C11—O1—C1—C10	79.3 (6)	C36—O3—C26—C35	82.7 (7)
C11—O1—C1—C2	−104.8 (6)	C36—O3—C26—C27	−100.6 (6)
C10—C1—C2—C3	−6.9 (8)	C35—C26—C27—C28	−3.2 (9)
O1—C1—C2—C3	177.3 (5)	O3—C26—C27—C28	−179.8 (5)
C10—C1—C2—C12	166.7 (5)	C35—C26—C27—C37	174.1 (5)
O1—C1—C2—C12	−9.1 (8)	O3—C26—C27—C37	−2.6 (8)
C1—C2—C3—C4	0.6 (8)	C26—C27—C28—C29	−2.4 (9)
C12—C2—C3—C4	−173.1 (5)	C37—C27—C28—C29	−179.6 (6)
C2—C3—C4—C5	−176.2 (5)	C27—C28—C29—C30	−176.3 (6)
C2—C3—C4—C9	4.3 (8)	C27—C28—C29—C34	3.8 (9)
C3—C4—C5—C6	−179.5 (5)	C34—C29—C30—C31	−1.9 (9)
C9—C4—C5—C6	0.0 (8)	C28—C29—C30—C31	178.2 (6)
C4—C5—C6—C7	0.3 (9)	C29—C30—C31—C32	1.3 (10)
C5—C6—C7—C8	0.0 (9)	C30—C31—C32—C33	−0.6 (11)
C6—C7—C8—C9	−0.5 (8)	C31—C32—C33—C34	0.5 (10)
C7—C8—C9—C4	0.8 (8)	C30—C29—C34—C33	1.7 (8)
C7—C8—C9—C10	−176.8 (5)	C28—C29—C34—C33	−178.3 (5)
C5—C4—C9—C8	−0.5 (7)	C30—C29—C34—C35	−179.8 (5)
C3—C4—C9—C8	179.0 (5)	C28—C29—C34—C35	0.2 (8)
C5—C4—C9—C10	177.1 (5)	C32—C33—C34—C29	−1.1 (9)
C3—C4—C9—C10	−3.3 (7)	C32—C33—C34—C35	−179.5 (6)
O1—C1—C10—C9	−176.7 (5)	O3—C26—C35—C34	−176.5 (5)
C2—C1—C10—C9	7.7 (8)	C27—C26—C35—C34	7.0 (8)
O1—C1—C10—C13	2.8 (8)	O3—C26—C35—C38	3.4 (8)
C2—C1—C10—C13	−172.8 (5)	C27—C26—C35—C38	−173.1 (5)
C8—C9—C10—C1	175.0 (5)	C29—C34—C35—C26	−5.4 (8)
C4—C9—C10—C1	−2.6 (7)	C33—C34—C35—C26	173.0 (5)
C8—C9—C10—C13	−4.5 (7)	C29—C34—C35—C38	174.7 (5)
C4—C9—C10—C13	177.9 (5)	C33—C34—C35—C38	−6.9 (8)
C3—C2—C12—Br1	−106.9 (6)	C28—C27—C37—Br3	98.3 (6)
C1—C2—C12—Br1	79.5 (6)	C26—C27—C37—Br3	−78.9 (6)
C1—C10—C13—C23	111.3 (6)	C26—C35—C38—C48	114.1 (6)
C9—C10—C13—C23	−69.1 (6)	C34—C35—C38—C48	−66.1 (6)
C24—O2—C14—C23	80.2 (7)	C49—O4—C39—C48	90.5 (6)
C24—O2—C14—C15	−103.8 (6)	C49—O4—C39—C40	−90.2 (6)
C23—C14—C15—C16	−5.0 (9)	C48—C39—C40—C41	−3.3 (8)
O2—C14—C15—C16	179.1 (5)	O4—C39—C40—C41	177.4 (5)
C23—C14—C15—C25	175.2 (6)	C48—C39—C40—C50	173.6 (5)
O2—C14—C15—C25	−0.7 (8)	O4—C39—C40—C50	−5.7 (7)
C14—C15—C16—C17	−2.1 (9)	C39—C40—C41—C42	−2.1 (8)
C25—C15—C16—C17	177.7 (5)	C50—C40—C41—C42	−178.9 (5)
C15—C16—C17—C18	−176.7 (6)	C40—C41—C42—C47	4.2 (8)
C15—C16—C17—C22	3.5 (9)	C40—C41—C42—C43	−176.8 (5)
C16—C17—C18—C19	178.9 (6)	C41—C42—C43—C44	179.0 (6)
C22—C17—C18—C19	−1.3 (9)	C47—C42—C43—C44	−2.0 (9)
C17—C18—C19—C20	−1.4 (10)	C42—C43—C44—C45	0.4 (9)
C18—C19—C20—C21	2.5 (9)	C43—C44—C45—C46	0.8 (9)
C19—C20—C21—C22	−0.8 (9)	C44—C45—C46—C47	−0.4 (9)
C20—C21—C22—C17	−1.8 (8)	C45—C46—C47—C42	−1.2 (8)
C20—C21—C22—C23	179.1 (5)	C45—C46—C47—C48	−178.5 (5)
C16—C17—C22—C21	−177.3 (5)	C41—C42—C47—C46	−178.7 (5)
C18—C17—C22—C21	2.9 (8)	C43—C42—C47—C46	2.3 (8)
C16—C17—C22—C23	1.7 (8)	C41—C42—C47—C48	−1.2 (8)
C18—C17—C22—C23	−178.0 (5)	C43—C42—C47—C48	179.8 (5)
O2—C14—C23—C22	−174.2 (5)	O4—C39—C48—C47	−174.7 (4)
C15—C14—C23—C22	10.1 (8)	C40—C39—C48—C47	6.0 (8)
O2—C14—C23—C13	6.5 (8)	O4—C39—C48—C38	5.1 (8)
C15—C14—C23—C13	−169.2 (5)	C40—C39—C48—C38	−174.2 (5)
C21—C22—C23—C14	170.7 (5)	C46—C47—C48—C39	173.7 (5)
C17—C22—C23—C14	−8.3 (8)	C42—C47—C48—C39	−3.7 (7)
C21—C22—C23—C13	−10.0 (8)	C46—C47—C48—C38	−6.1 (8)
C17—C22—C23—C13	171.0 (5)	C42—C47—C48—C38	176.6 (5)
C10—C13—C23—C14	111.8 (6)	C35—C38—C48—C39	114.0 (6)
C10—C13—C23—C22	−67.5 (7)	C35—C38—C48—C47	−66.3 (6)
C16—C15—C25—Br2	103.3 (6)	C41—C40—C50—Br4	97.5 (6)
C14—C15—C25—Br2	−76.9 (7)	C39—C40—C50—Br4	−79.2 (6)

### Hydrogen-bond geometry (Å, °)

**Table:**

*D*—H···*A*	*D*—H	H···*A*	*D*···*A*	*D*—H···*A*
C12—H12A···Br1^i^	0.99	2.91	3.867 (6)	164.
C37—H37B···O2^ii^	0.99	2.56	3.384 (8)	141.
C8—H8···O2	0.95	2.26	3.197 (7)	168.
C11—H11C···Br1	0.98	2.86	3.575 (7)	131.
C21—H21···O1	0.95	2.25	3.145 (7)	156.
C33—H33···O4	0.95	2.32	3.244 (7)	164.
C46—H46···O3	0.95	2.26	3.197 (7)	168.
C5—H5···Cg4^iii^	0.95	2.63	3.511 (7)	155.
C49—H49B···Cg7^iv^	0.98	2.73	3.505 (7)	136.
C43—H43···Cg1	0.95	2.71	3.625 (7)	161.

Symmetry codes: (i) −*x*+1, −*y*+1, −*z*+2; (ii) −*x*+1, −*y*+2, −*z*+1; (iii) −*x*+1, −*y*+2, −*z*+2; (iv) −*x*+2, −*y*+1, −*z*+1.
